# Pregnane X Receptor (PXR)-Mediated Gene Repression and Cross-Talk of PXR with Other Nuclear Receptors via Coactivator Interactions

**DOI:** 10.3389/fphar.2016.00456

**Published:** 2016-11-25

**Authors:** Petr Pavek

**Affiliations:** Department of Pharmacology and Toxicology and Centre for Drug Development, Faculty of Pharmacy in Hradec Kralove, Charles University in PragueHradec Kralove, Czechia

**Keywords:** PXR, nuclear receptor, gene regulation, metabolism, cross-talk

## Abstract

Pregnane X receptor is a ligand-activated nuclear receptor (NR) that mainly controls inducible expression of xenobiotics handling genes including biotransformation enzymes and drug transporters. Nowadays it is clear that PXR is also involved in regulation of intermediate metabolism through *trans*-activation and *trans*-repression of genes controlling glucose, lipid, cholesterol, bile acid, and bilirubin homeostasis. In these processes PXR cross-talks with other NRs. Accumulating evidence suggests that the cross-talk is often mediated by competing for common coactivators or by disruption of coactivation and activity of other transcription factors by the ligand-activated PXR. In this respect mainly PXR-CAR and PXR-HNF4α interference have been reported and several cytochrome P450 enzymes (such as CYP7A1 and CYP8B1), phase II enzymes (SULT1E1, Gsta2, Ugt1a1), drug and endobiotic transporters (OCT1, Mrp2, Mrp3, Oatp1a, and Oatp4) as well as intermediate metabolism enzymes (PEPCK1 and G6Pase) have been shown as down-regulated genes after PXR activation. In this review, I summarize our current knowledge of PXR-mediated repression and coactivation interference in PXR-controlled gene expression regulation.

## Introduction

Pregnane X receptor (PXR) is now accepted as a master transcription factor of xenobiotic- and drug-inducible expression of key genes that encode members of the phase I and phase II metabolic enzymes and drug transporters. Moreover, accumulating evidence suggests that PXR plays an integral role also in endobiotic metabolism by regulating important genes implicated in glucose, lipid, and bile acid metabolism. In addition, it was documented that PXR both induces as well as suppresses expression of numerous hepatic transcripts. The latest report shows that PXR up-regulates 164 genes, but down-regulates expression of 334 genes in primary human hepatocytes (Kandel et al., 2016). In this review I summarize well-documented examples of PXR-mediated *trans*-repression and coactivation interference in the PXR-mediated transactivation.

### Pregnane X Receptor

Nuclear receptors (NRs) form a super-family of transcription factors implicated in various physiological functions, from development, detoxification to homeostasis. Many NRs are ligand-activated transcription factors sharing a common evolutionary history and similar sequence features at the protein level, mainly in their DNA-binding domain (DBD), and to a lesser extent in ligand binding domains (LBD). PXR (or NR subfamily 1, group I, member 2, NR1I2), together with CAR (NR1I3), and vitamin D receptors (VDR, NR1I1), form a group I of the subfamily 1 of NRs (Moore et al., 2006; di Masi et al., 2009; Smutny et al., 2013).

Mouse PXR (mPXR) was first identified in 1998 by using an expressed sequence tag to screen a mouse liver library. It was found to be activated by derivatives of dexamethasone and pregnenolone (Kliewer et al., 1998). At the same time, the human steroid X receptor was cloned (Blumberg et al., 1998) and established to be the human homologue of mPXR involved in CYP450 3A4 regulation (CYP3A4) (Bertilsson et al., 1998; Lehmann et al., 1998).

Human PXR (hPXR) is the product of the *NR1I2* gene, which is located on chromosome 3, locus 3q11–q13.3. The *NR1I2* gene comprises 10 exons separated by nine intronic regions (Hustert et al., 2001; Zhang et al., 2001). PXR, like any other member in the NR super-family, is composed of the DBD, the H region, and the C-terminal LBD. PXR-DBD is involved in receptor dimerization and in the binding of specific DNA sequences. H region (or Hinge region) is a flexible domain that connects the DBD with the LBD. PXR heterodimerizes with RXRα to form a transcriptionally active complex (Blumberg et al., 1998; Lehmann et al., 1998).

The flexible ligand-binding pocket of PXR-LBD enables binding of a wide range of structurally unrelated endogenous and exogenous ligands. Watkins et al. (2001) first showed the crystal structure of the ligand-binding domain both alone and in complex with the PXR ligand SR12813. The PXR-LBD structure consists of a three-layered α-helical sandwich (α1–α3/α4–α5–α8–α9/α7–α10) and five-stranded antiparallel β-sheets (β1, β10, β2, β3, and β4). Interestingly, PXR-LBD contains an insert of approximately 60 residues which is unique within members of the NR super-family. This is the main reason for the larger cavity as well as the wider substrate diversity of PXR ligands. The *apo*-PXR binding cavity volume is approximately 1150 Å^3^; in the presence of ligands it can extend to 1290–1540 Å^3^ (Chrencik et al., 2005). Thus, the binding cavity volume is substantially larger than that of many other NRs. The ligand pockets of PXR, CAR as well as VDR are lined by mostly hydrophobic residues. The cavity of PXR is lined by 28 amino acids, of which eight have polar or charged side chains (Watkins et al., 2001; di Masi et al., 2009). The PXR-LBD ends with a short helix (αAF) which is critical for the structural organization of the AF-2 (the activation function 2) region to recruit transcriptional coregulators. In NR LBDs, the AF-2 region binds the Leu-Xxx-Xxx-Leu-Leu (LXXLL) motifs of transcriptional coactivators, and the Ile/Leu-Xxx-Xxx-Ile/Val-Ile motifs of corepressors (Lazar, 2003; Rosenfeld et al., 2006). The coactivator recruitment appears to play a central role in fixing ligands in the correct arrangement in the large PXR cavity after a coreppresor release.

Pregnane X receptor is primarily expressed in the liver, intestine, and to a lesser extend in the kidney. Expression of PXR/Pxr mRNA in other tissues including lung, stomach, peripheral blood monocytes, uterus, ovary, breast, adrenal gland, bone marrow, and some regions of the brain is minor (see the comprehensive review by Pavek and Dvorak, 2008). Mouse liver immunostaining suggests that mPXR is mainly located in the cytosol of untreated liver cells. Similar to CAR/mCar, mPxr forms a protein complex with cytoplasmic CAR retention protein (CCRP) and the heat shock protein 90 (hsp90), which retains the cytosolic localization of PXR. Upon ligand binding to mPxr, the Pxr dissociates from the multi-protein complex and translocates to the nucleus in primary mouse hepatocytes to activate gene transcription (Kawana et al., 2003; Squires et al., 2004). In contrast, nuclear localization of human PXR has been reported in mammalian tumor derived cell lines (Saradhi et al., 2005).

Pregnane X receptor was originally characterized as the key transcription factor that activates hepatic genes encoding drug-metabolizing enzymes and drug efflux transporters (Kliewer et al., 2002). PXR protects the body from harmful foreign toxicants or endogenous toxic substances by an autoregulation mechanism. PXR ligands activate a number of genes involved in their metabolism that in feedback manner contribute to their clearance. PXR has a wide spectrum of ligands belonging to drugs (such as antibiotic rifampicin, anticancer drugs tamoxifen and taxol, antihypertensive drug nifedipine, antifungal drug clotrimazole, or herbal antidepressant hyperforin), endogenous ligands (including steroids such as lithocholic acid) or products of gut microflora (di Masi et al., 2009; Smutny et al., 2013; Venkatesh et al., 2014).

Nowadays, it is clear that the xenobiotic-sensing PXR pathway regulates also energy metabolism, and reciprocally, the energy homeostasis affects drug metabolism. In the review, I focus on the PXR-mediated regulation^1^ of phosphoenolpyruvate carboxykinase (PEPCK) and glucose-6-phosphatase (G6Pase), two rate-limiting enzymes of hepatic gluconeogenesis; Hmgcs2, the key enzyme involved in ketogenesis; the active form of SREBP-1, which regulates genes required for sterol biosynthesis, fatty acid and lipid production and glucose metabolism; and lastly CYP7A1 and CYP8B1 enzymes critically involved in bile acid synthesis. PXR or its rodent orthologues have also been shown to be involved in heme, bilirubin and thyroxin clearance, in bone homeostasis and vitamin D metabolism. In addition, PXR activation is known to suppress the activity of NF-κB, which is the key regulator of inflammation and immune response (Xie et al., 2003; Zhou et al., 2009; Gao and Xie, 2012; Wang et al., 2012). These effects of PXR activation are, however, beyond the scope of the present review.

### PXR Coactivation

Coactivators and corepressors have been found critical for the function of DNA-binding transcription factors (TFs). Coactivator and corepressor proteins are components of multisubunit coregulator complexes involved in transcriptional gene regulation machinery (for a comprehensive review on coactivator/corepressors see Rosenfeld et al., 2006; Oladimeji et al., 2016).

PXR-LBD AF-2 region binds the LXXLL motifs of transcriptional coactivators as was shown with a 25 amino acid residue fragment of the human SRC-1. The motif forms hydrophobic contacts with the surface of hPXR in a groove composed of α3, α4, and AF-2 region. From the LBD side, PXR ligands are in direct contacts with αAF of AF-2 region (Xue et al., 2007). Both the SR12813 ligand and SRC-1 coactivator peptide in the crystal model stabilize the LBD of PXR. A charge clamp involving PXR residues Lys259 and Glu427 stabilizes the weak helix dipole at the C- and N-terminus of the LXXLL motif (Watkins et al., 2003; Xue et al., 2007).

SRC-1 was the first coactivator identified for hPXR (Kliewer et al., 1998; Lehmann et al., 1998). The members of the SRC family contain several conserved structural domains: a N-terminal basic helix-loop-helix-Per/ARNT/Sim (bHLH-PAS) domain, a central NR interaction domain (RID) with three LXXLL motifs, and two activation domains (AD1 and AD2) at the C-terminus. Once recruited to the target gene promoters by ligand-activated NRs, SRCs trigger the/an assembly of a multi-protein coactivator complex by further recruiting secondary coactivators and histone modifying enzymes to activation domains 1 and 2 (AD-1 and AD-2) such as CBP/p300, coactivator associated arginine methyltransferase 1 (CARM1) and protein arginine methyltransferase 1 (PRMT1). The formed transcriptional complex remodels transcriptionally inactive chromatin within the target gene and attracts components of the RNA polymerase II transcriptional complex. The AD-1 domain binds p300 and CBP, both of which are potent histone acetyltransferases (HATs) that remodel chromatin to allow accessibility for the transcription preinitiation complex. The AD-2 domain recruit protein arginine *N*-methyltransferase (PRMT) family members, such as CARM1 and PRMT1, which methylate residues of histone proteins and other chromatin-associated proteins (Szwarc et al., 2014; Wang et al., 2016).

The paradigm of NR action is that ligand binding enhances the receptor’s affinity for coactivator proteins, while decreasing its affinity for corepressors such as the silencing mediator of retinoid and thyroid receptors (SMRT, NR corepressor 2, NCoR2) and of the NR corepressor (NCoR, *NCOR1*), allowing further binding of the coactivators. Coactivators often have an intrinsic HAT activity, which weakens the association of histones to DNA, and therefore promotes gene transcription. On contrary, corepressors recruit histone deacetylases (HDACs), which strengthen the association of histones to DNA, and therefore repress gene transcription.

The mechanism of ligand-dependent activation of PXR significantly differs from that seen in many other NRs. Coactivation by SRC-1 stimulated after PXR activation has not been confirmed in some rigorous biophysical studies (Navaratnarajah et al., 2012). It was also demonstrated that PXR and SRC-1 interact in the absence of a PXR ligand, and further, that the interaction is strengthened by rifampicin (Saini et al., 2005; Li and Chiang, 2006; Rulcova et al., 2010; Krausova et al., 2011; Hyrsova et al., 2016). Moreover, no ligand activated release, or even stronger interaction with SMRTα has been reported for PXR (Takeshita et al., 2002; Mani et al., 2005; Li et al., 2009; Navaratnarajah et al., 2012; Hirooka-Masui et al., 2013). Nevertheless, also contradictory results have been published regarding SMRT and NCoR interaction with PXR (Ding and Staudinger, 2005b; Johnson et al., 2006). SMRT (or its overexpression) has been documented as the repressor of PXR-mediated transactivation (Takeshita et al., 2002; Mani et al., 2005; Johnson et al., 2006; Hirooka-Masui et al., 2013). In addition, repressed PXR by SMRT has been reported to inhibit the activation of *CYP24A1* gene by vitamin D receptor (Konno et al., 2009).

Recently, Masuyama et al. (2000) also found that PXR interacts with SRC-1 and NR interacting protein 1 (NRIP1, RIP140) in a ligand dependent manner. These data indicate that different ligands may specifically change the conformation of PXR-LBD resulting in different interaction with coactivators.

The interactions of co-factors with TFs are governed by post-translational modifications. Phosphorylation plays an important role in the regulation of NRs functions, enabling integration of different cellular and extracellular stimuli in their functions. PXR is phosphorylated by protein kinase A (PKA), resulting in strengthened interaction with SRC-1 and PBP coactivators (Ding and Staudinger, 2005a). In contrast, the activity of PXR can be repressed by the activation of protein kinase C (PKC) isoforms which alters the phosphorylation status of PXR and represses PXR-SRC-1 interaction, but strengthens PXR-NCoR interaction (Ding and Staudinger, 2005b). Therefore, the phosphorylation status of PXR can modulate coactivator/corepressor recruitment which could reflect the ligand-independent activation of PXR.

## NR Crosstalk Based on Coactivators Protein-Protein Interactions

As other NRs, PXR needs coactivators and corepressors for its transcriptional activity and for tuning of the tissue-, ligand-, and promoter (gene)-specific transactivation (Smutny et al., 2013). Coactivators and corepressors are common for the most of NRs and TFs. In addition, some NRs share the same response elements in transactivation of their target genes. This is the most strikingly evident in case of the *CYP3A4* gene, when PXR, CAR, and VDR share four different response elements in proximal promoter and two enhancer elements in gene- and tissue-specific manner (Pavek et al., 2010). This is the mechanistic molecular base for so called **cross-talk of NRs** in positive and negative transcriptional regulation, in forming negative feedback loops and in hierarchy of NRs in regulatory networks (Pascussi et al., 2008).

Recently, the competition for common coactivators emerged as an important process in the NR-mediated gene regulation. Competition for the common coactivators PGC-1α or GRIP-1 has been recently reported as a putative mechanism of crosstalk between CAR and the estrogen receptor (Min et al., 2002), HNF4α and PXR (Bhalla et al., 2004; Li and Chiang, 2005), and HNF4α and CAR (Miao et al., 2006). Competition for the common Src-1 coactivator has been reported for Pxr-Car crosstalk (Saini et al., 2005), as well as for Lxr and the retinoid-related orphan receptor α (Rorα) interaction (Wada et al., 2008); and considered as the underlying mechanisms in cases of Car-Lxrα/LXRα interaction (Zhai et al., 2010).

### Competition For Coactivators and Cross-Talk in PXR-Mediated Regulation of Intermediary Metabolism

Pregnane X Receptor has been shown to regulate glucose and lipid homeostasis during fasting and modifies the risk of hyperglycemia, diabetes, obesity, dyslipidemia, and hepatosteatosis. Key transcription factors and their cofactors in glucose and lipid homeostasis have been described to crosstalk with PXR regulation.

### CREB

In response to fasting and/or starvation, the liver increases the production of glucose by stimulating both gluconeogenesis and glycogenolysis. In the process glucagon up-regulates the transcription of the hepatic genes that encode rate-limiting enzymes of glucose homeostasis, such as the glucose-6-phosphatase catalytic subunit (**G6Pase**), or **PEPCK1** (phosphoenolpyruvate carboxykinase 1, PCK1). Glucagon stimulates PKA (cAMP-dependent protein kinase) that phosphorylates the CREB [CRE (cAMP response element)-binding protein]. The phosphorylation of CREB leads to the recruitment of HATs CBP/p300, binding of CREB to CREB response elements (CREs), and the activation of the CRE-bearing genes, such as those for *G6Pase* and *PEPCK1*, as well as PGC-1α. The coactivators linked to CREB transactivation (as well as FOXO1) include CBP/p300, CREB regulated transcription coactivator 2 (CRTC2), PGC-1α, and protein arginine methyltransferases (PRMTs) (Oh et al., 2013).

**FIGURE 1:**
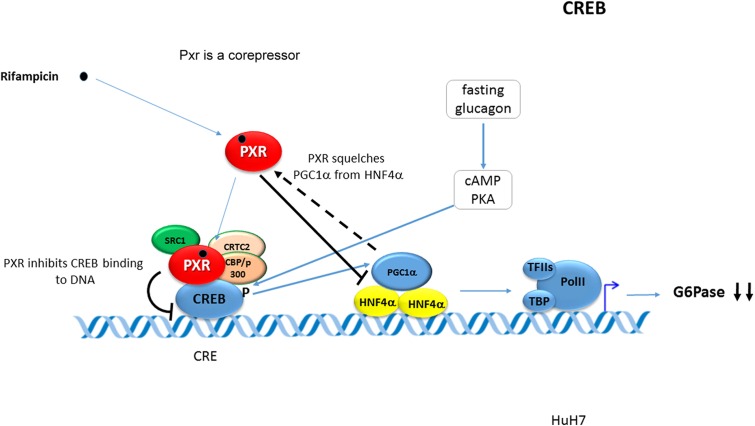
**Pregnane X Receptor represses the transcription of the G6Pase.** PXR represses the transcription of the *G6Pase* gene by inhibiting the DNA-binding ability of CREB to its response element CRE. PXR directly interacts with CREB. The figure has been drawn based on data by Kodama et al. (2007). Black bold arrows indicate effects of activated PXR on the *G6Pase* gene expression. Blue arrows indicate stimulation or activation. Dashed arrow indicates squelching (competition for a common coactivator). CRE -CREB response element.

It has been reported that rifampicin-activated PXR represses the transcription of the *G6Pase* gene by inhibiting the DNA-binding ability of CREB to its response element CRE and that direct interaction of PXR with CREB is involved (see **Figure 1**) (Kodama et al., 2007).

**FIGURE 2:**
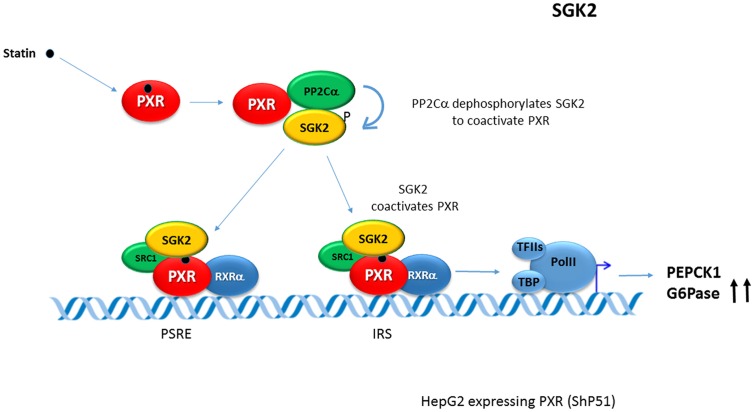
**Human-specific induction of PEPCK and G6Pase genes by PXR.** In the model, serum- and glucocorticoid-regulated kinase 2 (SGK2) has been found as an essential factor for PXR-induced glucose 6-phosphatase (G6Pase) up-regulation. Non-phosphorylated SGK2 co-activates PXR-mediated *trans*-activation of gluconeogenic genes in human liver cells, thereby enhancing gluconeogenesis and glucose production (Gotoh and Negishi, 2014, 2015). In the mechanism, activated PXR scaffolds the protein phosphatase 2C (PP2C) and SGK2 to stimulate PP2C to dephosphorylate SGK2. Dephosphorylated SGK2 co-activates PXR in the *trans*-activation of these *PEPCK1* and *G6Pase* genes. At the same time, ligand-activated PXR transactivates the expression of SGK2 (Gotoh and Negishi, 2014). Blue arrows indicate the effect of the activated PXR on tested genes expression. PSRE, PXR-SGK2 response elements; IRS, insulin response sequence (IRS).

In opposite to suppressive effects of PXR/mPxr activation on gluconeogenic genes via CREB signaling, human-specific induction of ***PEPCK*** and ***G6Pase*** genes have been described recently in rifampicin-treated HepG2 cells stably expressing human PXR. In these observations, serum- and glucocorticoid-regulated kinase 2 (SGK2) has been found as an essential factor for the PXR-induced *G6Pase* gene up-regulation. Non-phosphorylated SGK2 has been found to co-activate PXR-mediated *trans*-activation of gluconeogenic genes in human liver cells, thereby enhancing gluconeogenesis and glucose production (Gotoh and Negishi, 2014, 2015). In the mechanism the activated PXR scaffolds both the protein phosphatase 2C (PP2C) and SGK2 in order to stimulate PP2C to dephosphorylate SGK2. Dephosphorylated SGK2 co-activates PXR in the *trans*-activation of these genes (see **Figure 2**). At the same time, the ligand-activated PXR stimulates expression of the *SGK2* gene (Gotoh and Negishi, 2014). This finding of PXR-induced gluconeogenesis is consistent with the clinical observation that rifampicin can increase blood glucose level in humans (Hakkola et al., 2016; Rysa et al., 2013) even though the effect was attributed to the hepatic glucose transporter 2 (Glut2) mRNA down-regulation in subsequent experiments in rats (Rysa et al., 2013).

### FOXO1

The forkhead box O transcription factor FOXO1 is regarded as a master regulator of energy metabolism in numerous organs including the liver, pancreas, adipose tissue and skeletal muscle. FOXO1 regulates the transcriptional cascades controlling glucose and lipid metabolism. FOXO1 is an activator of gluconeogenic genes, such as ***PEPCK1***, ***G6P***, and ***insulin-like growth factor-binding protein 1*** (***Igfbp1***) via promoting the function of PGC-1α during fasting. Insulin inhibits FOXO1 activity leading to the repression of these genes. These gluconeogenic genes contain an insulin response sequence (IRS), which FOXO1 directly binds to, and activates them in the absence of insulin. Insulin triggers the phosphorylation of FOXO1 through the phosphatidylinositol 3-kinase (PI3K)-Akt pathway. Phosphorylation inactivates FOXO1 by decreasing its binding affinity to IRS of its target genes, which results in translocation of FOXO1 from the nucleus (see reviews by Kousteni, 2012; Oh et al., 2013).

**FIGURE 3:**
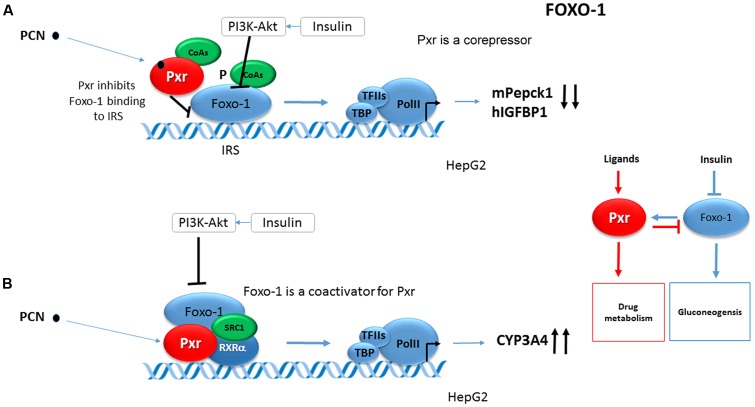
**Reciprocal crosstalk of Pxr and Foxo1.** **(A)** Foxo1 coactivates PXR in an insulin-PI3K-Akt-signaling dependent manner (Kodama et al., 2004). Foxo1 stimulates Pxr-mediated transactivation of *CYP3A4* gene reporter construct. Insulin as well as the constitutively active Akt abolishes the coactivation of mPxr by mouse Foxo1 in *CYP3A4* gene luciferase reporter construct activation in HepG2 cells. **(B)** PCN-activated mPxr inhibits mFoxo1 binding to IRS of human *IGFBP1* gene in EMSA assay (Kodama et al., 2004). Black bold arrows indicate effects of activated Pxr on gene expression. PCN, pregnenolone 16α-carbonitrile, a rodent specific ligand of Pxr

It has been shown that Foxo1 coactivates mouse Pxr (mPxr) in an insulin-PI3K-Akt-signaling dependent manner in the same way as CAR (Kodama et al., 2004). Foxo1 stimulates the Pxr-mediated transactivation of the ***CYP3A4*** gene reporter construct. Insulin as well as the constitutively active Akt abolished the coactivation of mPxr by mouse Foxo1 in CYP3A4 luciferase reporter construct activation in HepG2 cells (**Figure 3A**). At the same time PCN-activated Pxr inhibited the mouse Foxo1 binding to the IRS of human *IGFBP1* gene in EMSA assays. Based on the given data, Foxo1 and PXR were proposed to reciprocally coregulate their target genes (**Figure 3B**) (Kodama et al., 2004).

### FoxA2

FoxA2, a winged-helix/forkhead transcription factor, is the key regulatory factor for the normal development of endoderm-derived organs, such as the liver, pancreas, lungs, and prostate. FoxA2 is also important factor in the glucose and lipid metabolism control. FoxA2 activates gluconeogenic genes such as ***Pepck*** and ***G6p*** genes, as well as ***Cpt1a*** and ***Hmgcs2***. These enzymes are activated to increase the supply of glucose or ketone-bodies in fasting mouse liver (Wolfrum et al., 2004; Friedman and Kaestner, 2006).

**FIGURE 4:**
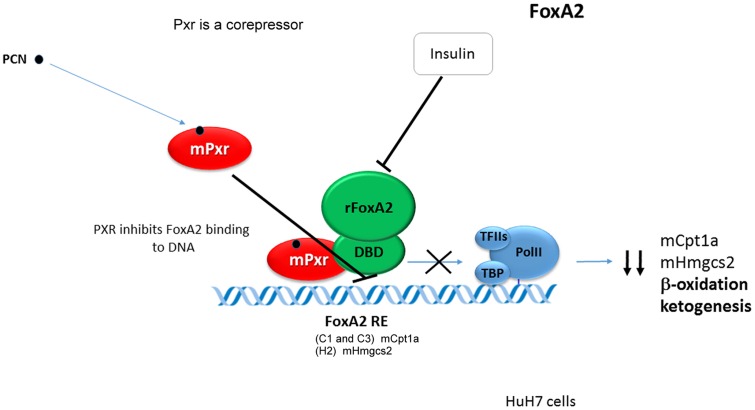
**Pregnane X Receptor cross-talks with the FoxA2 to repress the transcription of the Cpt1a and Hmgcs2 genes.** Mouse Pxr directly bounds to with the DBD of FoxA2 to inhibit its binding to the FoxA2 response elements and to repress *Cpt1a* and *Hmgcs2* genes transactivation (Nakamura et al., 2007). Black bold arrows indicate effects of activated Pxr on gene expression.

Pregnane X Receptor cross-talks with the FoxA2 to repress the transcription of the *Cpt1a* and *Hmgcs2* genes. mPXR was found to directly bind to the DBD of FoxA2 to inhibit its binding to the FoxA2 response elements in *Cpt1a* and *Hmgcs2* genes promoters (Nakamura et al., 2007) (see **Figure 4**).

### SREBP-1

Sterol regulatory element binding protein 1 is a lipogenic transcription factor of the basic helix-loop-helix family. Srebps are a group of transcription factors which activate an array of genes involved in the synthesis of cholesterol and triglycerides. Whereas Srebp-2 is mainly involved in cholesterol biosynthesis, Srebp-1a and Srebp1c, two isoforms encoded from different promoters, mainly activate genes involved in fatty acid and triglyceride synthesis. SREBP-1 binds to sterol regulatory elements (SREs) in promoters of lipogenic genes and induces fatty acid and triglyceride synthesis (Bakan and Laplante, 2012; Guo et al., 2014).

**FIGURE 5:**
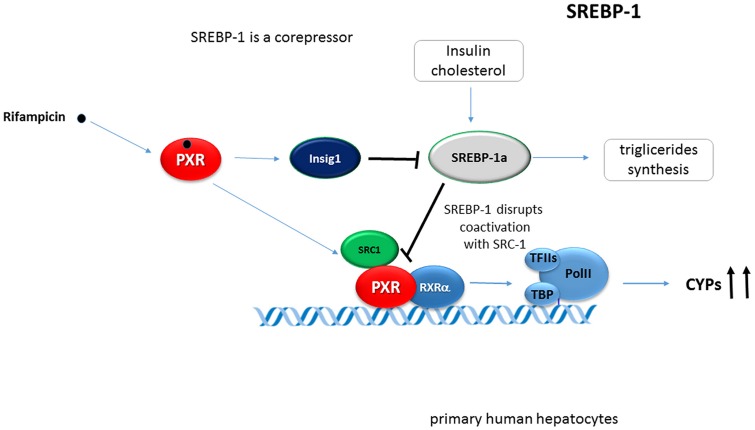
**SREBP-1 inhibits PXR-mediated transactivation of hepatic CYPs.** Activation of SREBP-1 by insulin in mouse liver and primary human hepatocytes inhibits the transcriptional effect of PXR due to SREBP-1 competing with coactivators such as SRC-1 (Roth et al., 2008b). PXR transcriptionally activates Insig-1 by binding to an enhancer sequence of the *Insig-1* gene reducing nuclear protein levels of the active form of Srebp-1. SREBP1a strongly interacts with PXR. Black bold arrows indicate the effect of activated PXR on genes expression.

It was observed that SREBP-1 attenuates drug-mediated induction of hepatic CYPs. The activation of SREBP-1 by insulin or low cholesterol levels in mouse liver and primary human hepatocytes inhibits the transcriptional effects of PXR (as well as of CAR) by SREBP-1 binding to and competing with coactivators such as SRC-1 (Roth et al., 2008b). Conversely, PXR transcriptionally activates Insig-1 by binding to an enhancer sequence of the *Insig-1* gene. Insig-1 in turn reduces the nuclear protein level of the active Srebp-1 (Roth et al., 2008a) (**Figure 5**).

## Competition for Coactivators and Cross-Talk in PXR-Mediated Regulation of CYP450 Enzymes Involved in Cholesterol/Bile Acid Metabolism and in Detoxification Mechanisms

Pregnane X Receptor is an important factor in controlling both cholesterol and bile acid synthesis, as well as in xenobiotic and endobiotic metabolism, respectively. CYP7A1, CYP8A1, and CYP3A4 genes and their animal orthologs are major target genes of the activated PXR/Pxr in these processes.

### PGC-1α

PPARgamma coactivator 1α is a key metabolic regulator of liver energy metabolism in fasting adaption and it was originally identified as a peroxisome proliferator-activated receptor-γ-interacting coactivator in brown adipose tissue. Numerous studies showed that PGC-1α is a versatile coactivator for numerous NRs implicated together in diverse biological functions including lipid and glucose metabolisms. In the liver PGC-1α has been shown to increase the HNF-4α-mediated transactivation of ***CYP7A1*** and together with FOXO1 and HNF4α controls the fasting-induced hepatic gluconeogenesis via ***PEPCK1*** and ***G6Pase*** genes. In addition, PGC-1α is involved in fatty-acid β-oxidation, ketogenesis and heme biosynthesis. In extrahepatic tissues, it controls adaptive thermogenesis, homocysteine metabolism, mitochondrial biogenesis, peripheral circadian clock, fiber-type switching in skeletal muscle and in heart development. The basal hepatic expression of PGC-1α is relatively low in fed conditions, but its expression is readily upregulated by fasting, glucagon and diabetes, mainly through an altered insulin–glucagon balance. PGC-1α cannot bind to DNA itself but functions as a coactivator via its LXXLL motif by interacting with a number of NRs and TFs, such as peroxisome proliferator-activated receptor alpha (PPARα), FOXO1, hepatocyte nuclear factor 4 alpha (HNF4α), mineralocorticoid (MR), glucocorticoid (GR), liver X receptors (LXR), the CAR, vitamin D receptor (VDR), or PXR. In addition, PGC-1α has a strong transcriptional activation domain at the N terminus, which interacts with several HAT complexes including CBP/p300 (Handschin, 2009; Liu and Lin, 2011).

Rifampicin, a prototype ligand for human PXR, is known to reduce hepatic bile acid levels in patients with cholestasis. Therefore, a functional cross-talk between PXR and HNF-4α, a key hepatic regulator of genes involved in bile acid synthesis including the cholesterol 7-alpha hydroxylase (***CYP7A1***) and sterol 12-alpha hydroxylase (***CYP8B1***) genes, has been studied. It was shown that PXR interacts with the coactivator PGC-1α through its C-terminal ligand binding domain in a rifampicin-dependent manner and that PGC-1α coactivates PXR transactivation (Bhalla et al., 2004; Li and Chiang, 2005, 2006; Hyrsova et al., 2016). Consistently, endogenous Pgc-1α from mouse liver extracts was found to bind to PXR, and recombinant PGC-1α directly interacts with PXR. In addition, rifampicin-dependent interaction of PXR with PGC-1α was shown in cells by co-immunoprecipitation and by intranuclear localization studies using confocal microscopy (Bhalla et al., 2004). Nevertheless, also conflicting results have been reported as regards significant rifampicin-mediated stimulation of PXR interaction with PGC-1α. No evidence of interaction was observed in GST pull-down assay and only a weak effect was seen in the mammalian two hybrid (M2H) assay (Li and Chiang, 2006).

**FIGURE 6:**
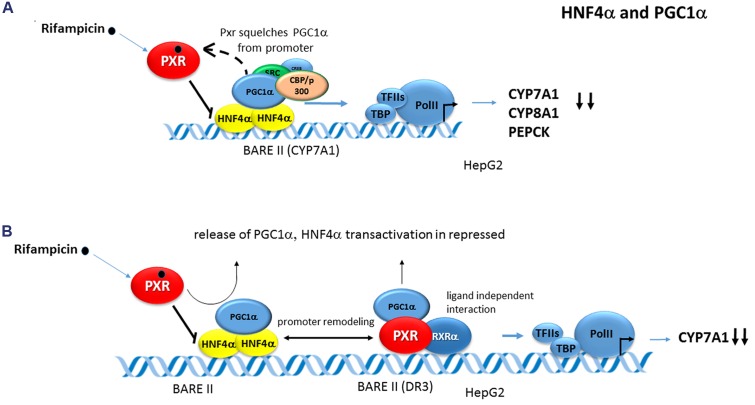
**Crosstalk of PXR with HNF4α in CYP7A1 and CYP8A1 regulation.** **(A)** Rifampicin did not inhibit HNF4α binding to the native promoters of *CYP7A1* and *CYP8B1* (and *PEPCK1)* genes but stimulates dissociation of PGC-1α from HNF4α by competing for binding PGC-1α to HNF4α. This leads to attenuation of HNF4α-mediated transactivation and down-regulation of the genes (Bhalla et al., 2004). **(B)** In another model, activated PXR triggers interaction of PXR with HNF4α in the context of promoter resulting in chromatin remodeling and release of PGC-1α. This again results in HNF4α-controlled expression attenuation (Li and Chiang, 2005). Black bold arrows indicate the inhibitory effects of activated PXR on genes expression. Dashed arrow indicates squelching (competition for a common coactivator).

PGC-1α at the same time coactivates and enhances the transcriptional activity of HNF-4α in the regulation of several liver-specific genes, including *CYP7A1*, *CYP8A1*, *SHP*, *OCT1 (SLC22A1)*, and *PEPCK1* (Bhalla et al., 2004; Li and Chiang, 2005, 2006; Hyrsova et al., 2016). This PGC-1α coactivation of HNF4α was reported to be suppressed by PXR ligands in an SHP-independent manner. In the case of ***CYP7A1*** and ***CYP8A1*** genes, rifampicin treatment did not inhibit HNF-4α binding to native promoters of these genes but resulted in dissociation of PGC-1α from HNF4α-formed transcription complex and subsequent gene repression (Bhalla et al., 2004). Most interestingly, the same effect was also observed in the *PEPCK1* regulation. The authors therefore proposed that PXR could be inhibitory in the process by competing for PGC-1α binding to HNF4α that dominantly controls the high hepatic expression of *CYP7A1, CYP8A1*, and *PEPCK* genes (Bhalla et al., 2004) (see also chapter HNF4α, **Figure 6A**).

A slightly different model of PXR-HNF4α crosstalk in *CYP7A1* gene repression has been proposed by Li and Chiang (Li and Chiang, 2005). In their experiments employing M2H assay and ChiP, rifampicin enhanced PXR interaction with HNF4α in the context of *CYP7A1* gene promoter, but reduced PGC-1α interaction with HNF4α resulting in overall *CYP7A1* repression (see also chapter HNF4α, **Figure 6B**).

**FIGURE 7:**
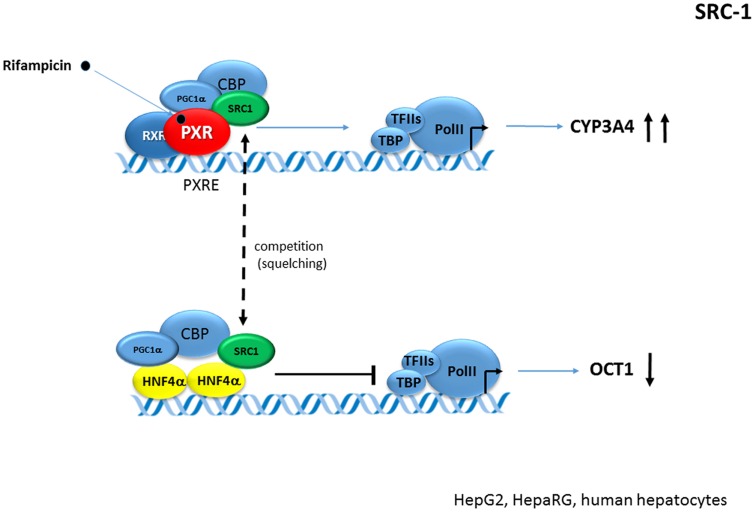
**Crosstalk of PXR and HN4α in OCT1 gene repression.** Activated PXR competes and deplete (“squelches”) the SRC-1 coactivator from HNF4-mediated transactivation of *OCT1* (*SLC22A1*) gene. OCT1 is dominantly regulated by HNF4α in the liver (Hyrsova et al., 2016). Dashed arrow indicates the squelching (competition for common coactivator). Black bold arrows indicate the effects of activated PXR on genes expression

In a recent study, we examined the suppressive effect of activated PXR on **OCT1** (SLC22A1) expression. OCT1 is one of the most tightly controlled genes with HNF4α transactivation. We rather observed competition for SRC-1 than PGC-1α coactivator in PXR-HNF4α interaction, thereby suggesting a gene(promoter)-specific crosstalk of PXR-HNF4α in the case of *OCT1* gene regulation (Hyrsova et al., 2016) (**Figure 7**).

### HNF4α

Hepatocyte nuclear factor 4α is a master transcriptional activator for a large number of genes in hepatocytes and pancreatic cells. HNF4α belongs to the “orphan” NRs (it is classified as NR2A1), although fatty-acid CoA thioesters have been proposed as its ligands. Mutations in this gene have been associated with the monogenic autosomal dominant non-insulin-dependent diabetes mellitus type I (MODY 1, maturity onset diabetes of the young). HNF4α also belongs among the so called liver-enriched transcription factors controlling liver physiology, differentiation and drug-metabolism enzymes expression (Jover et al., 2009). By using a combination of ChiPs and promoter microarrays, 910 genes in hepatocytes and 758 genes in pancreatic islets were regulated by HNF4α in regulatory circuits together with HNF1α and HNF6 transcription factors (Odom et al., 2004). HNF4α is essential for cholesterol and glucose/energy metabolism because it is a key factor for the basal hepatic expression of *CYP7A1*, *CYP8B1*, *G6Pase*, and *PEPCK* genes, respectively.

These genes all contain functional HNF4α-binding sites in their promoter, and mutation of these sites substantially disrupt promoter activation. HNF4α binds as a homodimer mainly to DR1 response elements. Such DR1 motifs have been found in the bile acid responsive element (BARE) II region at -148 to -129 in the human *CYP7A1* gene promoter and at +198 to +227 of the human *CYP8B1* gene promoter. The promoter of human PEPCK gene also contains a functional HNF4α-binding site at -431 to -418 (Watt et al., 2003; Crestani et al., 2004).

**FIGURE 8:**
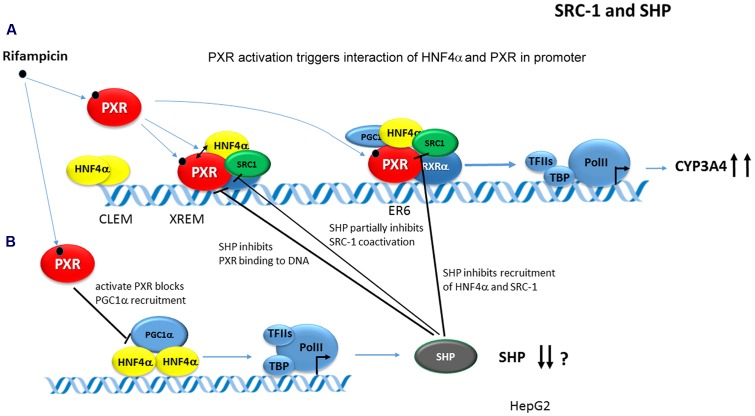
**Model of PXR-mediated transactivation of CYP3A4 gene.** In the model, PXR ligands stimulate PXR/Pxr binding to DNA and interaction of PXR with HNF4α. PXR-HNF4α-SRC-1 complex transactivates *CYP3A4* gene promoter **(A)**. PXR at the same time trans-represses *SHP* gene expression. SHP interferes with PXR-HNF4α-SRC-1 coactivation and with PXR-DNA interaction **(B)** (Ourlin et al., 2003; Li and Chiang, 2006).

Hepatocyte nuclear factor 4α augments the PXR/Pxr-mediated transactivation of the human ***CYP3A4*** and mouse ***Cyp3a11*** genes (Tirona et al., 2003; Li and Chiang, 2006). This enhancement was proposed through *cis*-acting HNF4α-binding sites in the proximal promoter and at the far upstream enhancer region for the *CYP3A4* gene (Tirona et al., 2003; Matsumura et al., 2004; Pavek et al., 2010) even though an HNF4α-RE independent mechanism has also been proposed (Li and Chiang, 2006). Rifampicin strongly stimulates PXR and HNF4α interaction in *CYP3A4* gene transactivation, which is further augmented by PGC-1α and SRC-1 coactivators, but inhibited by Small heterodimer partner (SHP, NR0B2) (Tirona et al., 2003; Li and Chiang, 2006). ChiPs revealed that the rifampicin-activated PXR recruits HNF4α and SRC-1 (but not PGC-1α) to the *CYP3A4* gene chromatin. In *CYP3A4* transactivation, SHP, PXR, and HNF4α have been proposed to interact and compete for binding to each other (Li and Chiang, 2006). Concomitantly, PXR has been proposed to inhibit SHP promoter activity and to repress *SHP* gene transcription by disrupting PGC-1α coactivation of HNF4α (Li and Chiang, 2006) (**Figure 8**).

In the case of ***CYP7A1*** gene promoter, HNF4α directly binds with PXR and both NRs are strongly co-activated by PGC-1α (Li and Chiang, 2005, 2006). With respect to PXR-mediated *CYP7A1* gene repression, two theories regarding the mechanism have been postulated based on the PXR-HNF4α-PGC-1α crosstalk. Bhalla et al. (2004; Dr. Jongsook Kim Kemper’s group) proposed that PXR competes for the binding of PGC-1α with HNF4α in *CYP7A1* gene regulation and squelches PGC-1α from HNF4α/DNA complex (**Figure 6A**). The group of Dr. Chiang proposed that the activation of PXR by rifampicin promotes PXR interaction with HNF4α in the *CYP7A1* gene promoter, but blocks PGC-1α interaction mainly with HNF4α and to a lesser extend with PXR. This results in the inhibition of *CYP7A1* gene expression dominantly transactivated by HNF4α-PGC-1α (Li and Chiang, 2005) (**Figure 6B**). The latter authors also argue that “squelching” of the common coactivator PGC-1α is an unlikely mechanism since PGC-1α mostly interacts with PXR in a ligand-independent manner.

Pregnane X Receptor activation by rifampicin was also found to repress the estrogen sulfotransferase 1E1 (***SULT1E1***) gene. Mechanistic studies showed that activated PXR displaces HNF4α bound to the PXR-responsive enhancer of *SULT1E1* gene resulting in promoter remodeling, histone 3 deacetylation and repressed expression (Kodama et al., 2011).

### SRC-1

Steroid receptor coactivator 1 is a well-known coactivator with the conserved N-terminal basic helix-loop-helix-Per/ARNT/Sim (bHLH-PAS) domain, a central NR interaction domain (RID) with three LXXLL motifs, and two activation domains (AD1 and AD2) at the C-terminus. SRC-1 interacts with many NRs including PXR, LXRα, CAR, FXR, HNF4α, GR etc. It was shown that PXR and SRC-1 interact in the absence of a PXR ligand and the interaction is strengthened by rifampicin (Saini et al., 2005; Li and Chiang, 2006; Rulcova et al., 2010; Krausova et al., 2011; Hyrsova et al., 2016), although SRC-1 was proposed to bind to PXR much weaker that PGC-1α (Li and Chiang, 2005).

Interesting results have been obtained with *Pxr*-null, *Car*-null and double-KO mice. In *Pxr*-null mice, *Car* target genes *Mrp2*, *Mrp3*, *Ugt1a1*, *Oatp4*, and *Gsta2* were up-regulated. A detailed investigation has shown that unliganded Pxr may attract coactivators such as Src-1 from Car to dominate over Car and to control the constitutive activity of Car in detoxification enzymes regulation (Saini et al., 2005). Based on this, the ligand-free Pxr can suppress both the constitutive and ligand-induced activity of Car by competing for common coactivator Src-1 in a target gene specific manner. This finding also highlights a regulatory hierarchy of Pxr/Car (PXR/CAR) cross-talk in the regulation of common target detoxifying enzymes (Saini et al., 2005).

The antidiabetic drug metformin was reported to suppress PXR-regulated transactivation of *CYP3A4* gene (Krausova et al., 2011). Metformin did not affect PXR expression, instead it disturbed PXR interaction with SRC-1 (Krausova et al., 2011). Since there is some analogy with the regulation of PXR and PGC-1α interaction by metformin-induced SIRT1, we can speculate that SIRT1 can play a role in the process (Hakkola et al., 2016).

### SHP

The small (or short) heterodimer partner (SHP) is a NR (NR subfamily 0, group B, member 2) encoded by the *NR0B2* gene in humans. SHP is unusual as a NR in that it lacks a DNA binding domain. No endogenous ligand has been found, therefore SHP belongs to the “orphan” subfamily. SHP executes its regulatory function through protein–protein interactions as a coreppresor of numerous NRs (including PXR, CAR, LXRs, PPARs, HNF4α, LRH-1, GR, TRβ, RARα, FXR, ERs, ERRs etc.), TFs (such as FOXO1, C/EBPα, NF-κB etc.) or kinases (such as C-jun or Smad3) (Zhang et al., 2011). SHP represses the transcriptional activities of its target proteins by utilizing two functional LXXLL-related motifs in the LBD domain. The binding of SHP to TFs/NRs either competes or dissociates coactivators on the AF-1/2 domains from the receptors. SHP is thus involved in bile acid, cholesterol, triglyceride, glucose, and drug metabolism. SHP mainly plays important role in the negative regulation of the conversion of cholesterol to bile acids via FXR, as well in regulating the expression of genes playing roles in bile acid transport (BSEP, NTCP), lipid metabolism (SREBP1C), and gluconeogenesis (PEPCK, G6Pase) (Zhang et al., 2011; Zou et al., 2015).

It was shown that SHP inhibits PXR-mediated transactivation of the *CYP3A4* gene by interfering with PXR binding to promoter response elements (Ourlin et al., 2003; Pavek et al., 2012; Smutny et al., 2014). However, this finding has been questioned by Li and Chiang (Li and Chiang, 2006). Instead, they proposed that HNF4α and SHP compete for binding to PXR in *CYP3A4* gene transactivation after rifampicin treatment. In addition, SHP partially blocks PXR-SRC-1 (but not PXR-PGC-1α) interaction in *CYP3A4* gene regulation (Li and Chiang, 2006). Interestingly, rifampicin strongly enhanced PXR-SHP interaction in M2H and GST pull-down assays (Li and Chiang, 2006). In addition, it was shown that the activated PXR *trans*-represses SHP expression, which is dominantly controlled by HNF4α-PGC-1α regulation, by blocking PGC-1α recruitment to *SHP* gene promotor chromatin (Li and Chiang, 2006). By this mechanism, PXR may concomitantly inhibit SHP gene transcription and maximizes the PXR-mediated induction of the *CYP3A4* gene in human livers (Li and Chiang, 2006) (see **Figure 8**).

### SIRT1

SIRT1 (silent mating type information regulation 2 homolog 1) also named as NAD^+^-dependent deacetylase sirtuin-1, is a protein that is encoded by the SIRT1 gene in humans. SIRT1 is a deacetylase protein which is both catalytically activated by increased NAD^+^ level and also transcriptionally induced during fasting. SIRT1, a mammalian ortholog of the yeast Sir2 protein, belongs into the class III of HDAC that has been reported to deacetylate many target proteins including some NRs, either activating or repressing their functions. Sirtuin 1 is a key metabolic/energy sensor and mediates homeostatic responses to caloric restriction. Accumulating evidence indicates that Sirtuin 1 is a master regulator that controls hepatic lipid metabolism. During fasting conditions, SIRT1 deacetylates and alters the expression and the activities of key transcriptional regulators involved in hepatic lipogenesis, β-oxidation, and cholesterol/bile acid metabolism (Moore et al., 2012; Kemper et al., 2013). In addition, SIRT1 deacetylates PGC-1α and thus enhances its ability to coactivate gluconeogenic genes (Rodgers et al., 2005).

**FIGURE 9:**
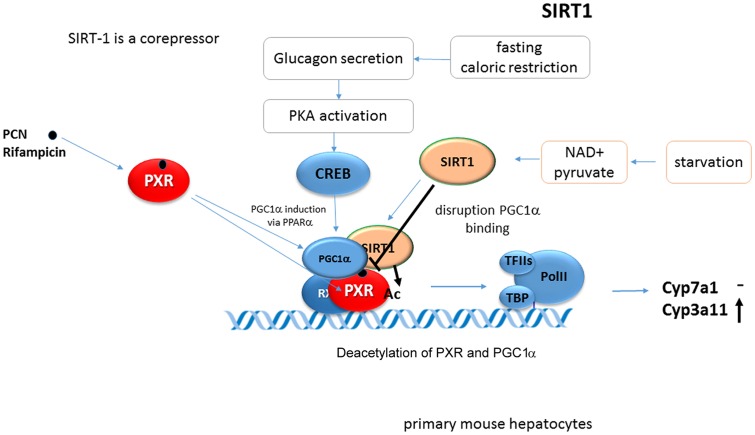
**SIRT1 represses Pxr-PGC-1α coactivation in Cyp3a11 gene regulation.** SIRT1 binds to PXR and deacetylate PXR after its ligand-dependent activation. Sirt1 interferes with Pxr-Pgc-1α coactivation in *Cyp3a11* gene regulation (Biswas et al., 2011; Buler et al., 2011).

SIRT1 is one of two major regulators of hepatic energy homeostasis (together with PGC-1α) involved in PXR signaling. SIRT1 binds and deacetylates PXR after the ligand-dependent activation of PXR (Biswas et al., 2011; Buler et al., 2011). Interestingly, Buler et al. (2011) found PGC-1α-mediated regulation of PXR expression. SIRT1 was also shown to interfere with PCN-induced Pxr coactivation by Pgc-1α in *Cyp3a11* gene transactivation (**Figure 9**). Thus SIRT1 and PGC-1α fasting-activated pathways differentially affect PXR/Pxr-mediated function.

### RXRα

Pregnane X Receptor heterodimerizes with the NR retinoid X receptor α (RXRα, NR2B1), encoded by the *RXRA* gene (Blumberg et al., 1998; Kliewer et al., 1998). Similarly, other NRs such as CAR, FXR, LXR, and PPARs form heterodimers with hetero-oligomeric partner RXRα. Therefore, it is logical to suppose crosstalk due to the competition for the common heteropartner. However, no NR-NR interactions have been reported at the level of RXR crosstalk between these receptors in the literature. It was shown that ligand-activated LXRα transactivates *CYP3A4* gene expression but suppresses the PXR-dependent transcription of *CYP3A4* through known PXR-responsive elements dNR1 and eNR3A4. However, the amount of RXRα was not found as the limiting factor in *CYP3A4* transcription after simultaneous activation of PXR and LXRα (Watanabe et al., 2013). We can thus speculate that there is sufficiently high amount of RXRα in hepatocytes to sustain parallel heterodimerization with several activated NRs. We should also take into account that PXR forms functional homodimer, which again suggests that RXRα amount may not be a limiting factor for PXR activity (Noble et al., 2006).

Some retinoids and rexinoids, ligands of RXRα, significantly induce the PXR/RXR-mediated transactivation (Wang et al., 2006; Pettersson et al., 2008). Interestingly, rexinoids can antagonize PXR activation by rifampicin due to the reduced binding of PXR/RXR to PXR response elements. In addition, rexinoids, bexarotene (LGD1069), and LG100268 can stimulate protein degradation of both PXR and RXR (Pettersson et al., 2008). Therefore, ligand-dependent PXR-RXR interactions may have an effect on PXR target genes expression.

### NF-κB

Proinflammatory stimuli down-regulate CYP expression and drug-metabolizing activities in the liver.

NF-κB is the key regulator of inflammation and immune responses. The NF-κB family comprises five members, namely p65 or Rel A, Rel B, c-Rel, p50, and p52. NF-κB normally resides in the cytoplasm bound to the protein inhibitor of NF-κB (IκB). Activating signals, such as pro-inflammatory cytokines lead to phosphorylation and degradation of IκB, thus allowing NF-κB to translocate to the nucleus. In the nucleus, NF-κB directly regulates the transactivation and expression of its target genes.

Signaling mediated by lipopolysaccharide (LPS) and cytokines, such as IL-1 and TNFα, leads to the activation of NF-κB. Activated NF-κB was shown to repress PXR activation and the PXR-mediated induction of several CYPs. Pascussi et al. first have shown that the proinflammatory cytokine interleukin 6 (IL-6) down-regulates PXR mRNA in primary human hepatocytes and inhibits the rifampicin-mediated induction of the PXR target CYPs genes such as *CYP2B6, CYP2C8/9*, and *CYP3A4*. However, PXR activity itself was not affected by the IL-6 in reporter assays (Pascussi et al., 2000).

**FIGURE 10:**
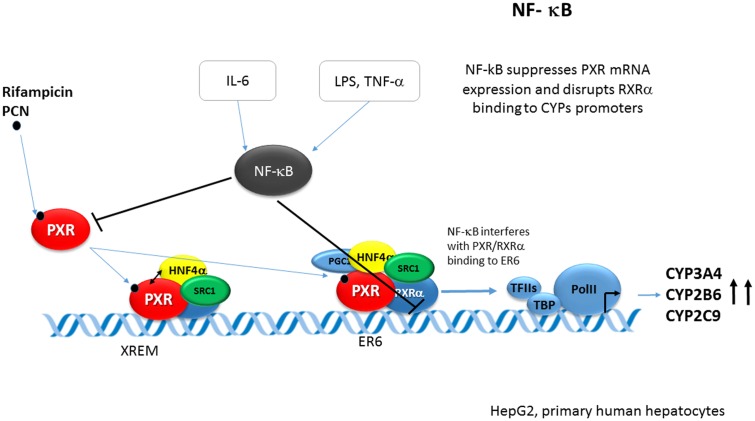
**Crosstalk of NF-κB p65 and PXR-RXRα in PXR-mediated CYPs regulation.** Proinflammatory cytokine interleukin 6 (IL-6) down-regulates PXR mRNA in primary human hepatocytes. In addition, activation of NF-κB by either LPS or TNF-α leads to PXR suppression through interaction of NF-κB p65 and the PXR-RXRα heterodimer. NF-κB p65 directly binds to RXRα DBD and thus disrupts the association of the PXR/RXRα complex with the PXR responsive sequence ER6 of the *CYP3A4* gene (Pascussi et al., 2000; Gu et al., 2006).

Gu et al. (2006) reported that the activation of NF-κB by either LPS or TNF-α leads to PXR suppression through the interaction of NF-κB p65 and PXR-RXRα heterodimer. NF-κB p65 disrupted the association of the PXR/RXRα complex with the PXR responsive sequences ER6 in electrophoretic mobility shift and ChiPs. This interference has been explained by the direct binding of NF-κB p65 to the RXRα DBD in the GST pull-down assay (**Figure 10**).

Zhou et al. (2006) further corroborated the NF-κB-PXR crosstalk and reported that NF-κB activation inhibits hPXR activation, and that inhibition of NF-κB potentiates PXR activation. In addition, they showed that NF-κB target genes are up-regulated and small bowel inflammation is significantly increased in Pxr^-/-^ mice.

In addition to NF-κB signaling, both protein kinase C and A (PKC and PKA) signaling is involved in repressing *CYP3A* gene expression by affecting the PXR activity during inflammation. Mechanistically, posttranslational modifications of PXR by the kinase signaling are involved. I refer to other reviews for more details (Smutny et al., 2013; Oladimeji et al., 2016).

Thus, the reciprocal crosstalk between PXR and NF-κB is the proposed mechanism for the anti-inflammatory function of PXR and down-regulation of PXR target CYPs during inflammation.

## Discussion and Conclusion

In the review, I comprehensively summarize our current knowledge about the molecular mechanisms of: (i) competition for coactivator binding to PXR, (ii) coactivation of PXR by other transcription factors or NRs leading to NRs cross-talk, (iii) signaling and posttranslational modification of PXR that impairs its coactivation and (iv) *trans*-repression of TFs and NRs by PXR. All these processes form the NR-signaling network that enables at the same time sensing to caloric restriction or to toxic injury. Some of the mechanisms confirm our view on the hierarchy of xenobiotic and endobiotic metabolism regulation and propose novel targets for drug development. However, considering the number of NRs, coactivators, corepressors and signaling pathways that orchestrate transcriptome and proteome regulation, it is clear that we have discovered only a minor part of the network.

Pregnane X Receptor plays both positive and negative roles in regulating numerous genes involved in homeostasis and detoxification. Indeed, the latest report shows that PXR down-regulates expression of twice as many genes than it induces in primary human hepatocytes (Kandel et al., 2016). According to current knowledge, *SULT1E1*, *SHP*, *HNF4α*, *OCT1*, and *FOXO1* genes are the only candidates that have been reported as repressed genes of activated PXR in detailed mechanistic studies (Li and Chiang, 2006; Zhou et al., 2006; Kodama et al., 2011; Kodama et al., 2015). In the case of these genes, however, protein-protein interactions of coactivators with NRs or TFs take place and no direct *trans*-repression via a *cis*-acting PXR repression element has been reported so far.

Therefore, a better understanding of the coactivator/coreppresor relationships in PXR-mediated gene regulation may help us do delineate the regulation of other genes repressed by activated PXR in PXR expressing tissues.

## Author Contributions

The author confirms being the sole contributor of this work and approved it for publication.

## Conflict of Interest Statement

The author declares that the research was conducted in the absence of any commercial or financial relationships that could be construed as a potential conflict of interest.

## Footnotes

1 In all NRs, m and h denote mouse and human, respectively.

## Abbreviations

CAR: constitutive androstane receptor
ChiP: Chromatin immunoprecipitation assay
CREB: cAMP-response element-binding protein
CYP450: cytochrome P450
CYP3A4: cytochrome P4503A4, CYP7A1, cholesterol 7α-hydroxylase
DR-1: direct repeat separated by one nucleotide
FoxA2: forkhead factor 2
FOXO1: forkhead box protein O1
FXR: farnesoid X receptor
HNF4α: hepatocyte nuclear factor 4α
Hmgcs2: mitochondrial 3-hydroxy-3-methylglutarate-CoA synthase 2
GST: glutathione *S*-transferase
M2H: mammalian two hybrid assay
NR: nuclear receptor
OCT1: organic cation transporter 1
PGC-1alpha: PPARgamma coactivator 1α
PXR: pregnane X receptor
PGC-1α: peroxisome proliferator-activated receptor gamma, coactivator 1 α, (PPARGC1A)
RXRα: Retinoid X Receptor Alpha
SGK2: serum/glucocorticoid regulated kinase 2
SLC22A1: solute carrier family of transporter A 1
SRC-1: steroid receptor coactivator
SREBP-1: Sterol regulatory element binding protein 1
